# *Mycobacterium tuberculosis*-Specific IL-21^+^IFN-γ^+^CD4^+^ T Cells Are Regulated by IL-12

**DOI:** 10.1371/journal.pone.0147356

**Published:** 2016-01-19

**Authors:** Li Li, Yuxia Jiang, Suihua Lao, Binyan Yang, Sifei Yu, Yannan Zhang, Changyou Wu

**Affiliations:** 1 Institute of Immunology, Zhongshan School of Medicine, Key Laboratory of Tropical Disease Control Research of Ministry of Education, Sun Yat-sen University, Guangzhou, China; 2 Wuhan Institute for Tuberculosis Control, Wuhan Pulmonary Hospital, Wuhan, China; 3 Chest Hospital of Guangzhou, Guangzhou, China; Colorado State University, UNITED STATES

## Abstract

In the current study of *Mycobacterium tuberculosis* (MTB)-specific T and B cells, we found that MTB-specific peptides from early secreted antigenic target-6 (ESAT-6) and culture filtrate protein-10 (CFP-10) induced the expression of IL-21 predominantly in CD4^+^ T cells. A fraction of IL-21-expressing CD4^+^ T cells simultaneously expressed Th1 cytokines but did not secrete Th2 or Th17 cytokines, suggesting that MTB-specific IL-21-expressing CD4^+^ T cells were different from Th1, Th2 and Th17 subpopulations. The majority of MTB-specific IL-21-expressing CD4^+^ T cells co-expressed IFN-γ and IL-21^+^IFN-γ^+^CD4^+^ T cells exhibited obviously polyfunctionality. In addition, MTB-specific IL-21-expressing CD4^+^ T cells displayed a CD45RO^+^CD62L^low^CCR7^low^CD40L^high^ICOS^high^ phenotype. Bcl-6-expression was significantly higher in IL-21-expressing CD4^+^ T cells than IL-21^-^CD4^+^ T cells. Moreover, IL-12 could up-regulate MTB-specific IL-21 expression, especially the frequency of IL-21^+^IFN-γ^+^CD4^+^ T cells. Taken together, our results demonstrated that MTB-specific IL-21^+^IFN-γ^+^CD4^+^ T cells from local sites of tuberculosis (TB) infection could be enhanced by IL-12, which have the features of both Tfh and Th1 cells and may have an important role in local immune responses against TB infection.

## Introduction

Tuberculosis (TB) is one of the most ancient diseases of mankind and currently remains a leading cause of death from infectious disease worldwide [1–3]. The incidence of TB has increased over the past few years for reasons such as inadequate preventative efforts, incorrect or inappropriate medication, the emergence of drug-resistant strains of *Mycobacterium tuberculosis* (MTB) and the prevalence of human immunodeficiency virus (HIV) infection [4–6].

Cell-mediated immunity is known to be crucial for protection against TB and most studies have shown that CD4^+^ and CD8^+^ T cells are essential for protective immunity [7–10]. We have been committed to studying MTB-specific effector and memory CD4^+^ T cells, including Th1, Th17, and Th22 cells [11,12], and we have identified the epitopes, functions and regulation of CD8^+^ T cells against MTB infection [13,14]. Recently, we found that pleural fluid cells (PFCs) secrete IL-21 following stimulation with specific peptides.

IL-21, a potent immunomodulatory cytokine, has pleiotropic effects on both innate and adaptive immune responses [15–17]. Owing to the broad cellular distribution of the IL-21 receptor, IL-21 exerts pleiotropic effects on the immune system [16,18]. The role of IL-21 in sustaining and regulating T cell, B cell, and NK cell responses during autoimmune diseases, chronic infectious diseases and immunodeficiency diseases has recently come into focus [17,19,20].

It has been reported that follicular helper T (Tfh) cells, Th17 cells, NKT cells, Th1 cells and Th2 cells can produce IL-21, although Tfh cells have the closest relationship with IL-21 [21–24]. In addition, activated human dendritic cells have been shown to induce naïve CD4^+^ T cells to become IL-21-expressing Tfh-like cells through IL-12 [25]. Tfh cells in humans were initially described in 2000 and 2001, when several groups reported that a large proportion of CD4^+^ T cells in tonsils have a unique phenotype and express high levels of chemokine (C-X-C motif) receptor 5 (CXCR5) [24]. Currently, Tfh cells are considered to be a distinct CD4^+^ T cell type and they are important for protective immunity [24,26]. Those cells are characterized by expression of the transcription factor B-cell lymphoma 6 (Bcl-6), production of high amounts of the B-cell stimulatory cytokine IL-21, and increased levels of CXCR5, inducible costimulator (ICOS) and programmed death 1 (PD-1) [24,26,27].

In the current study, we tried to define the relationship between MTB-specific IL-21-expressing cells and Tfh cells. We conducted studies to determine the immunophenotypical characteristics, functional properties and regulatory factors of MTB-specific IL-21-expressing CD4^+^ T cells. Our data demonstrated that MTB-specific IL-21-expressing CD4^+^ T cells are present at local sites of infection in patients with tuberculous pleurisy (TBP) and these cells may play an important role in local cellular immunity against TB infection.

## Results

### MTB-specific peptides induce IL-21 production by PFCs

**Fig 1:**
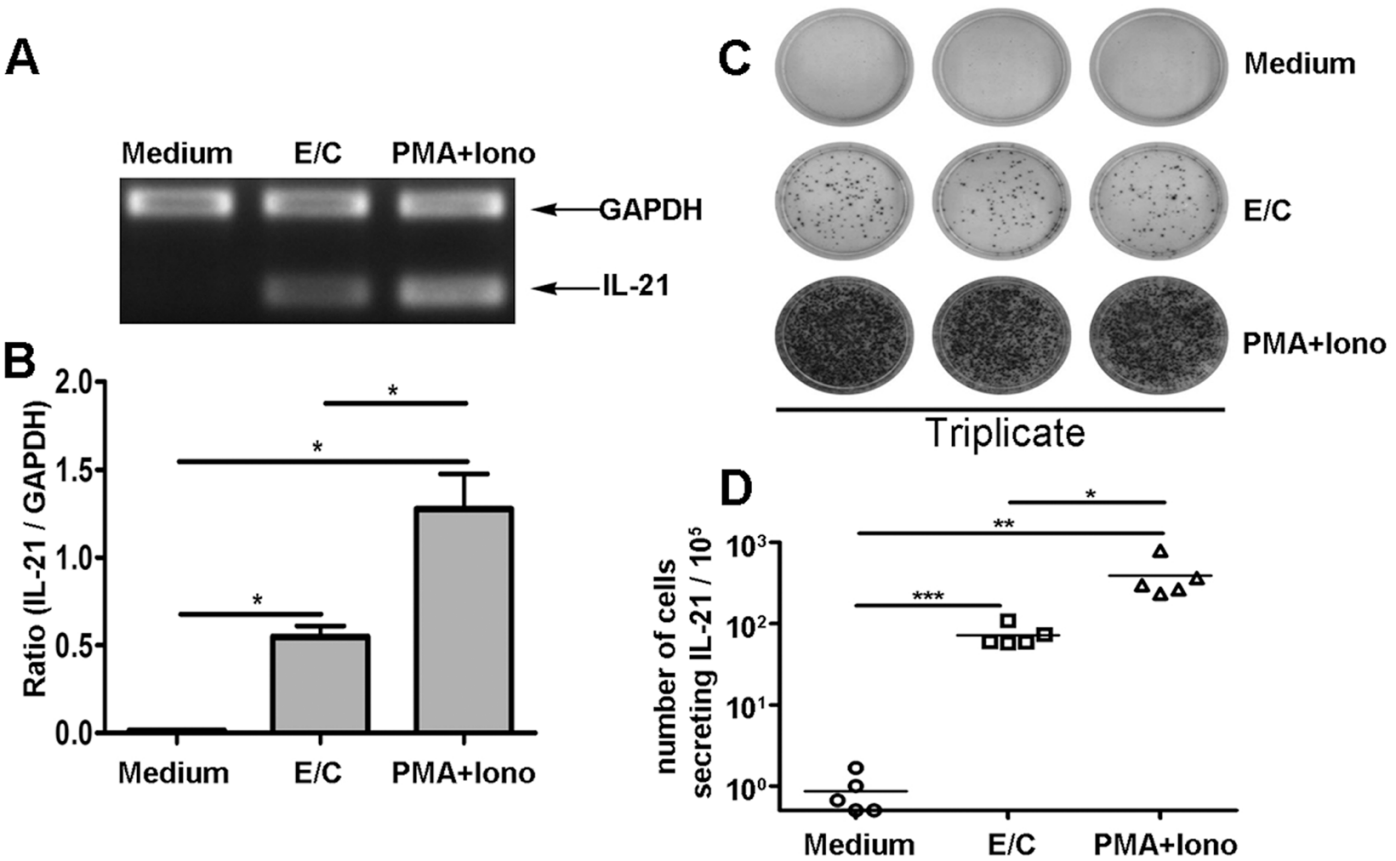
ESAT-6/CFP-10 (E/C) peptides induced IL-21 production at the levels of mRNA and protein by PFCs in tuberculous pleurisy. (A,B) PFCs were cultured in the presence of medium alone, with E/C peptides or with PMA plus ionomycin for 12 h, after which IL-21 and GAPDH mRNA levels were determined by RT-PCR (upper panel). The graph (lower panel) shows the ratio of IL-21 over GAPDH, calculated according to the relative intensities of the bands revealed under UV illumination with Bio-1D software. Data are representative of five separate experiments with similar results. (C) A representative data of ELISPOT results is shown. Data are representative of five separate experiments with similar results. (D) The frequency of IL-21-producing cells was enumerated by ELISPOT assay (n = 5). Each dot represents one donor. Horizontal bars represent the mean value. *p<0.05; **p<0.01; *** *p*<0.001.

To determine whether the MTB-specific peptides ESAT-6 and CFP-10 (E/C) induce IL-21 production, PFCs were cultured in the presence of medium alone, E/C peptides, or PMA plus ionomycin. RT-PCR results revealed that E/C peptides induce markedly higher levels of IL-21 mRNA transcription than cultures with medium alone. As expected, PMA plus ionomycin also induced significantly high levels of IL-21 (Fig 1A and 1B). To further analyze the frequency of IL-21-producing cells, an enzyme-linked immunospot (ELISPOT) assay was conducted. IL-21^+^ spots were not detectable without stimulation. E/C peptides, however, elicited a strong antigen-specific T cell response with an average of 71 spot-forming cells (SFCs) (range, 57–108 SFCs), which was significantly higher than in medium alone (Fig 1C and 1D). PMA plus ionomycin induced a stronger response. Altogether, these results indicated that E/C peptides induced IL-21 production by PFCs at both mRNA and protein levels.

### CD4^+^ T cells from PFCs express IL-21 following stimulation with E/C peptides

**Fig 2:**
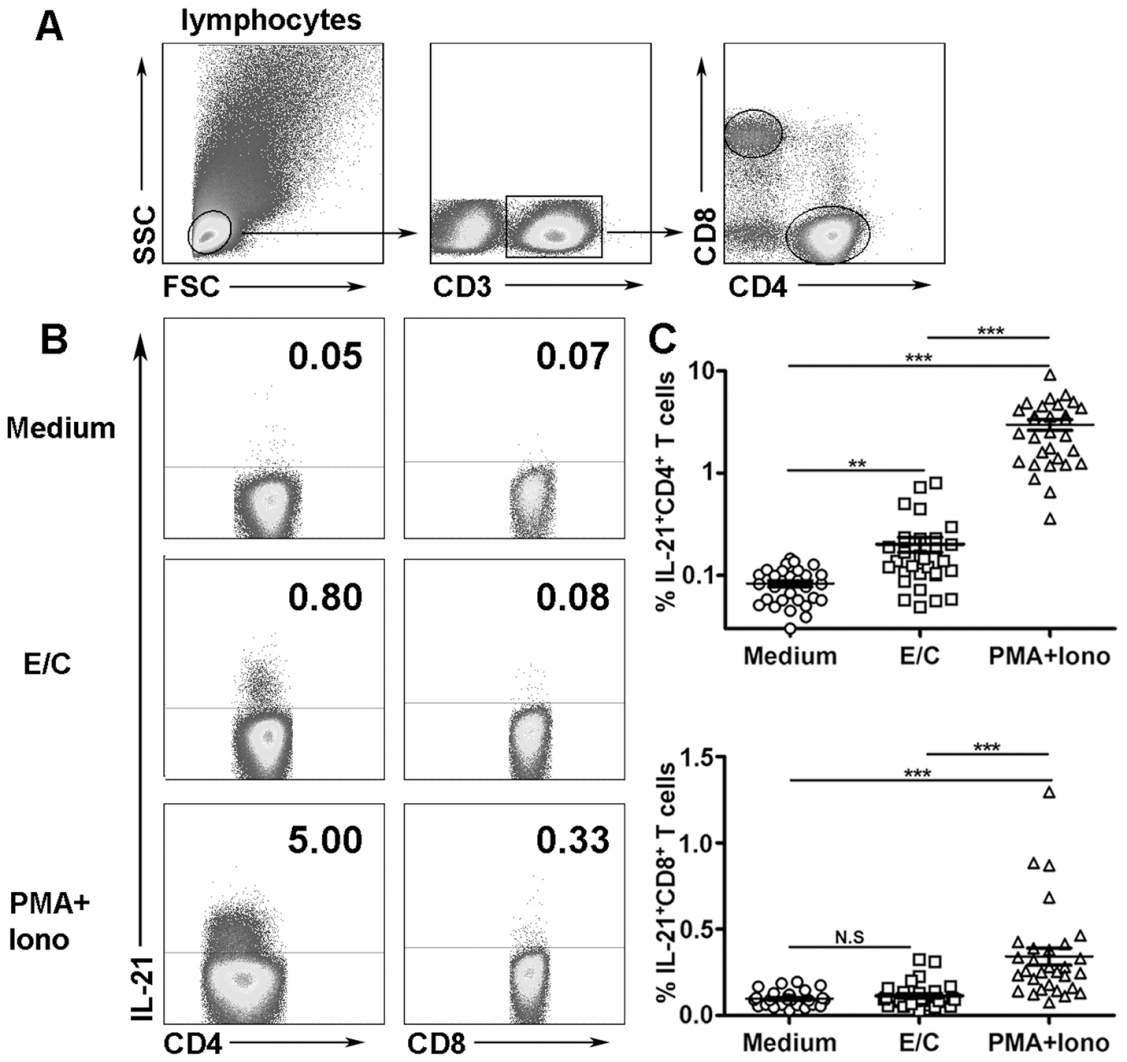
E/C peptides induced IL-21 expression by CD4^+^ T cells from PFCs. (A) Flow cytometric gating strategy used for analysis of lymphocytes from PFCs. (B) PFCs were stimulated in the presence of medium alone, with E/C peptides, or with PMA plus ionomycin. The expression of IL-21 was evaluated by FACS. Representative dot plots of thirty independent experiments are shown. (C) Statistical results of IL-21 expression by CD4^+^ and CD8^+^ T cells from PFCs are shown as the mean ± SEM. **p<0.01; *** *p*<0.001; N.S., not significant.

To further determine which subsets of cells produce IL-21, fluorescence-activated cell sorting (FACS) was conducted. The results showed a very low frequency of IL-21 expression by PFCs in the presence of medium alone. Following stimulation with E/C peptides, CD4^+^ T cells, but not CD8^+^ T cells, expressed IL-21 (Fig 2A and 2B). Notably, PMA plus ionomycin induced strong IL-21 expression in CD4^+^ T cells, which was consistent with the results from ELISPOT. Statistical analysis indicated that E/C peptides induced significantly higher percentages of IL-21-expressing CD4^+^ T cells than medium alone (Fig 2C, n = 30, *p*<0.01), but no significant difference was observed in CD8^+^ T cells. In addition, PMA plus ionomycin induced significantly high levels of IL-21 expression by CD4^+^ and CD8^+^ T cells.

### The subset of IL-21-expressing CD4^+^ T cells is different from Th1, Th2, and Th17 subpopulations

**Fig 3:**
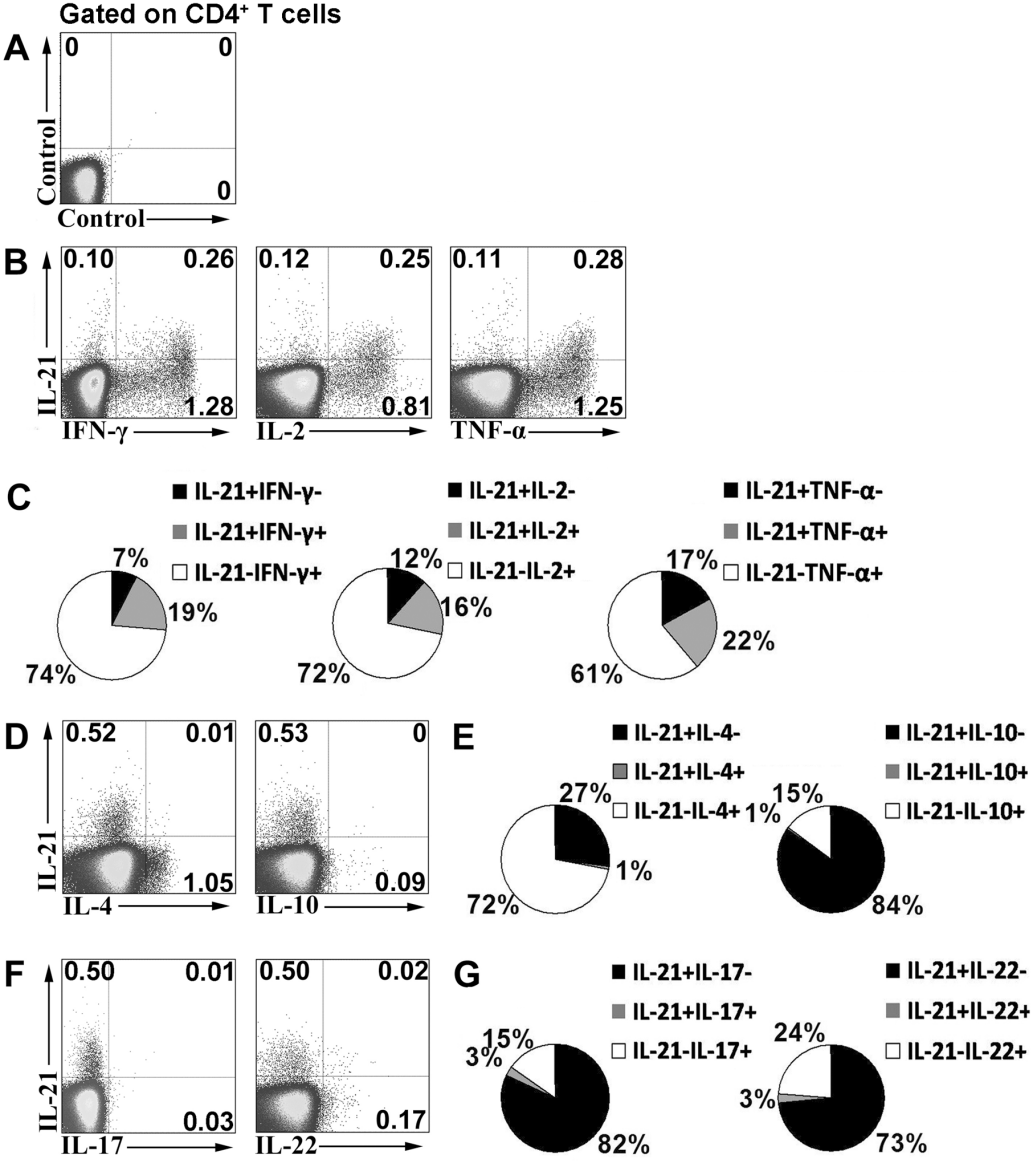
Correlation of IL-21 expression with Th1, Th2 or Th17 cytokines following E/C peptides stimulation. PFCs were stimulated with E/C peptides. The expression of cytokines was determined by FACS. (A) An isotype control is shown. (B) Expression of IL-21 and the Th1 cytokines, IFN-γ, IL-2, and TNF-α was assessed by FACS. Numbers in quadrants indicate percentages of cells in each population. (C) Data are quantified and presented in a pie chart; each slice of the pie represents the fraction of the mean value of a given quadrant. Independent experiments were repeated at least eight times. (D) Expression of IL-21 and the Th2 cytokines, IL-4 and IL-10, was assessed by FACS. Data are representative of seven separate experiments. (E) Corresponding statistical results are shown in pie charts. (F) IL-21, IL-17 and IL-22 levels were assessed by FACS. Data are representative of seven separate experiments. (G) Corresponding pie charts are shown.

**Fig 4:**
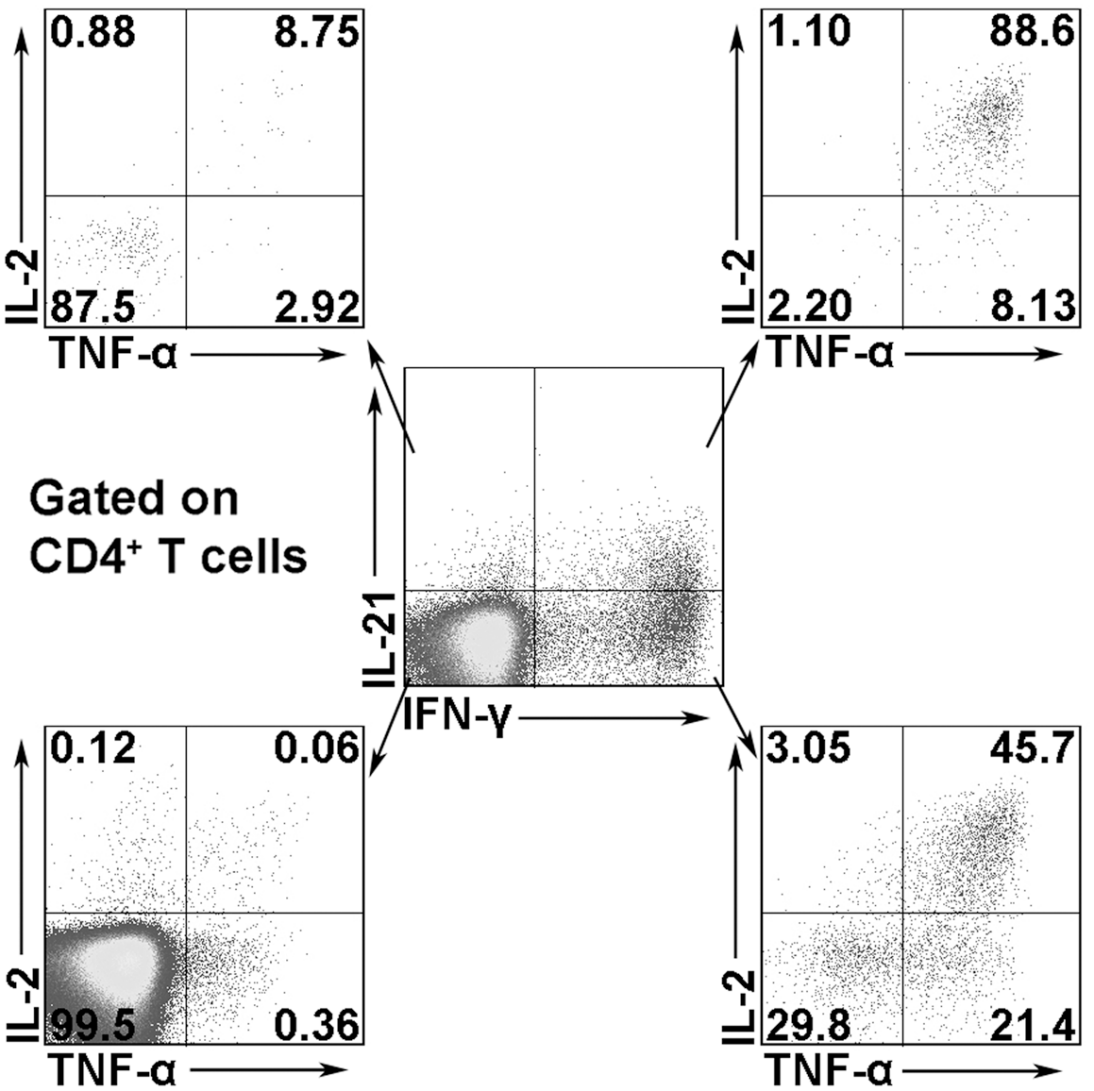
Polyfunctional IL-21^+^IFN-γ^+^CD4^+^ T cells following E/C peptides stimulation. PFCs were stimulated with E/C peptides. CD4^+^ T cells were gated in lymphocytes from PFCs. The expression of IL-21 and IFN-γ was detected by FACS. The expression of IL-2 and TNF-α within each cell subset was analyzed. Representative data of six independent experiments are shown.

To determine whether MTB-specific IL-21-expressing cells are related to Th1, Th2, and Th17 cell populations, PFCs were stimulated with E/C peptides, and the expression of cytokines was analyzed. In the correlation between IL-21 and IFN-γ expression, cytokine-expressing cells could be clearly divided into three subsets: IFN-γ^+^IL-21^-^, IL-21^+^IFN-γ^+^, and IL-21^+^IFN-γ^-^ cells. A fraction of IL-21-expressing cells simultaneously expressed IFN-γ. A similar correlation was observed between IL-21 and IL-2 or TNF-α (Fig 3A and 3B). Statistical analysis showed that IFN-γ single-positive cells had the largest proportion of cytokine-secreting cells, followed by IL-21^+^IFN-γ^+^ cells and IL-21 single-positive cells. Similar distributions were observed between IL-21 and IL-2 or TNF-α, as shown in the pie charts in Fig 3C. Interestingly, IL-21-expressing cells did not express IL-4 and IL-10 (Fig 3D). The pie charts in Fig 3E show the distributions between IL-21 and IL-4 or IL-10. Moreover, IL-21-expressing cells did not express IL-17 and IL-22 (Fig 3F). Corresponding statistical results are shown Fig 3G. Taken together, these results indicate that a subset of human IL-21-expressing CD4^+^ T cells co-expressed Th1 cytokines. Meanwhile, IL-21-expressing cells were distinct from Th2, Th17, and Th22 cells based on cytokine expression. To further evaluate the polyfunctionality of IL-21^+^IFN-γ^+^ cells, CD4^+^ T cells were divided into four subsets: IFN-γ^+^IL-21^-^, IL-21^+^IFN-γ^+^, IL-21^+^IFN-γ^-^ and IL-21^-^IFN-γ^-^ T cells according to the production of IL-21 and IFN-γ. The expression of IL-2 and TNF-α within these distinct cell subsets was further analyzed. The results indicated that IL-21^+^IFN-γ^+^ T cells co-expressed high levels of IL-2 and TNF-α compared to IL-21^+^IFN-γ^-^ T cells, suggesting the obviously polyfunctionality of these cells (Fig 4).

### CD4^+^IL-21^+^ T cells are effector or effector memory T cells

**Fig 5:**
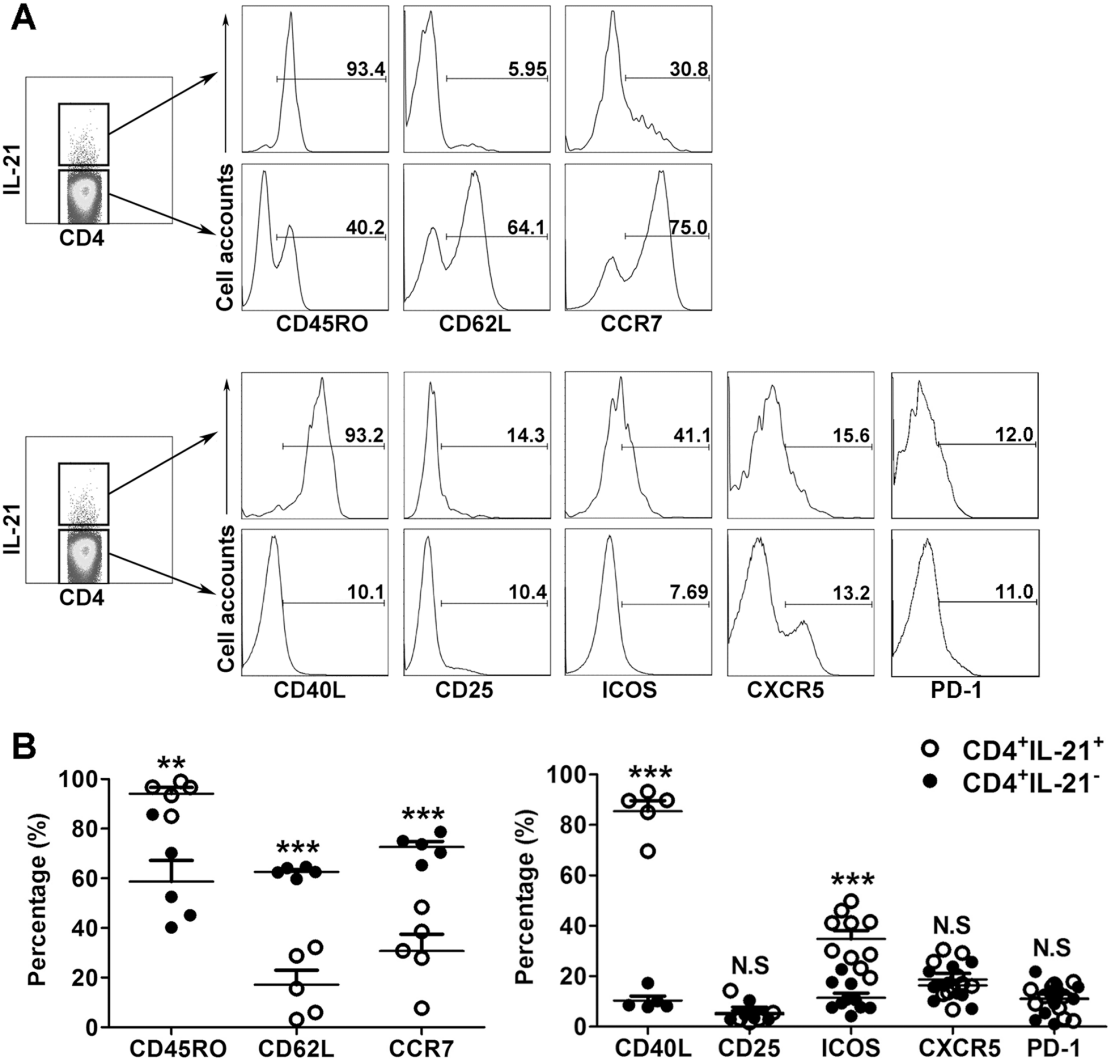
Phenotype of CD4^+^IL-21^+^ and CD4^+^IL-21^-^ T cells. PFCs were stimulated with E/C peptides. IL-21 and surface markers expressed by CD4^+^ T cells were evaluated by FACS. CD4^+^IL-21^+^ and CD4^+^IL-21^-^ T cells were gated. (A) The expression of the typical memory T cell markers, CD45R0, CD62L and CCR7, and the activation markers, CD40L, CD25, ICOS, CXCR5 and PD-1, by CD4^+^IL-21^+^ and CD4^+^IL-21^-^ T cells are shown. Representative flow cytometric graphs are shown. (B) Summary data of the frequency of CD45R0, CD62L, CCR7, CD40L, CD25, ICOS, CXCR5 and PD-1 expression by CD4^+^IL-21^+^ and CD4^+^IL-21^-^ T cells. Data shown are the mean ± SEM from five to ten independent experiments. **p<0.01; *** *p*<0.001; N.S., not significant.

**Fig 6:**
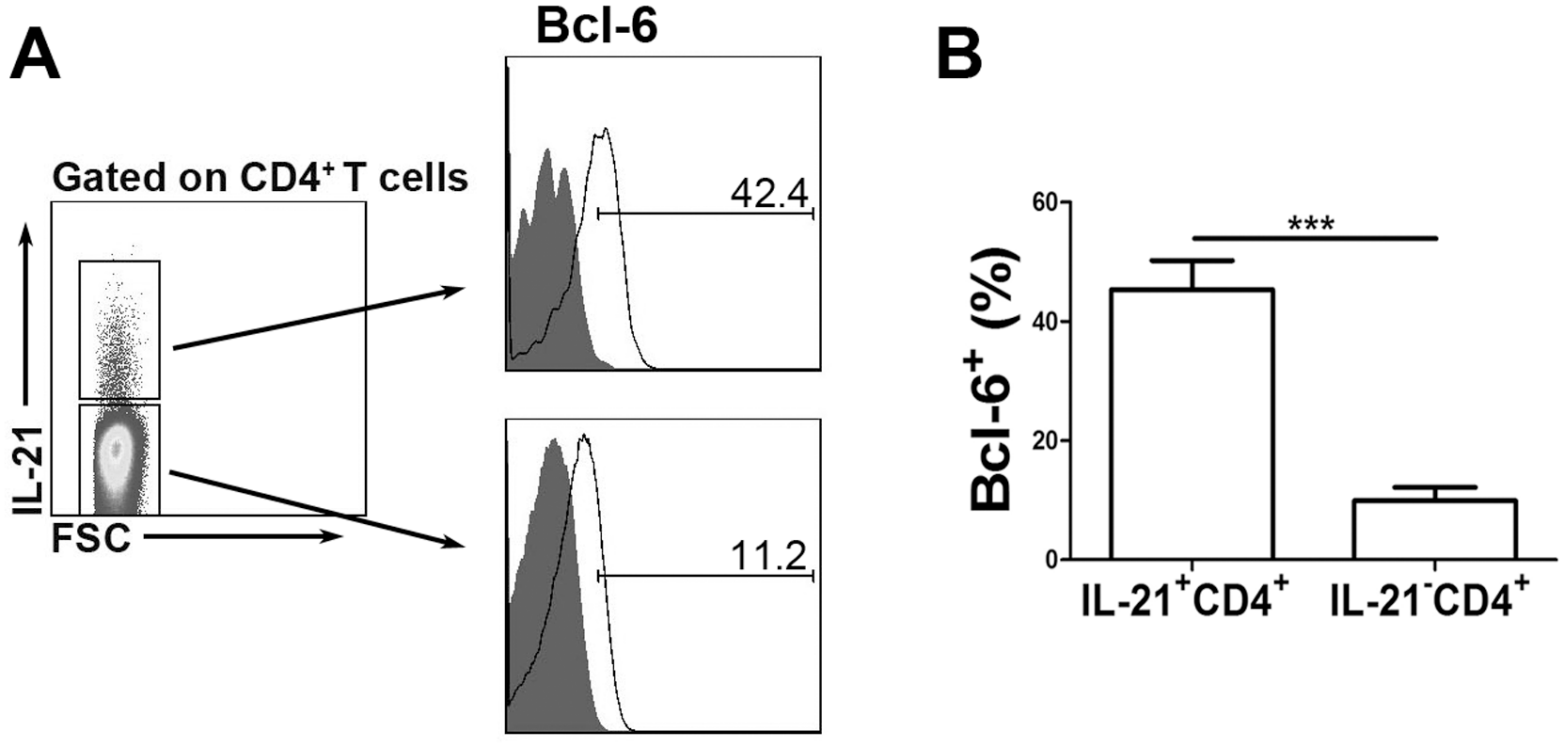
Bcl-6 expression was significantly higher on CD4^+^IL-21^+^ T cell subset than CD4^+^IL-21^-^ T cells. PFCs were stimulated with E/C peptides. IL-21 and Bcl-6 expression by CD4^+^ T cells was evaluated by FACS. (A) CD4^+^IL-21^+^ and CD4^+^IL-21^-^ T cells were gated. The expression of Bcl-6 on CD4^+^IL-21^+^ and CD4^+^IL-21^-^ T cells is shown. Data are representative of five independent experiments. (B) Statistical results of Bcl-6 expression on IL-21^+^ and IL-21^-^ CD4^+^ T cells. *** *p*<0.001.

We next investigated the phenotype of the CD4^+^IL-21^+^ T cells with respect to memory and activation markers. As shown in Fig 5A, the CD4^+^IL-21^+^ T cells were found to be predominantly CD45RO^+^, thereby exhibiting a memory cell phenotype. In contrast, the CD4^+^IL-21^-^ T cells consisted of a significantly low percentage of CD45RO^+^ cells. We also found that CD4^+^IL-21^+^ T cells expressed significantly lower levels of CD62L and CCR7 than CD4^+^IL-21^-^ T cells. We also examined the expression of activation markers, such as CD40L, CD25 and ICOS. Significantly higher levels of CD40L and ICOS expression were detected on CD4^+^IL-21^+^ T cells compared to CD4^+^IL-21^-^ T cells. However, no significant difference was observed in terms of CD25 expression by CD4^+^IL-21^+^ and CD4^+^IL-21^-^ cells (Fig 5B). CXCR5 and PD-1 were reported to be important markers for Tfh cells. However, we did not find any differences in terms of CXCR5 and PD-1 expression between CD4^+^IL-21^+^ and CD4^+^IL-21^-^ cells. Bcl-6 is the key transcription factor for Tfh cell differentiation and function. Therefore, we also assessed the expression of Bcl-6. The results indicated that CD4^+^IL-21^+^ cells had significantly higher expression of Bcl-6 than CD4^+^IL-21^-^ cells (Fig 6).

### IL-12 can up-regulate IL-21^+^IFN-γ^+^CD4^+^ T cells induced by MTB-specific peptides

**Fig 7:**
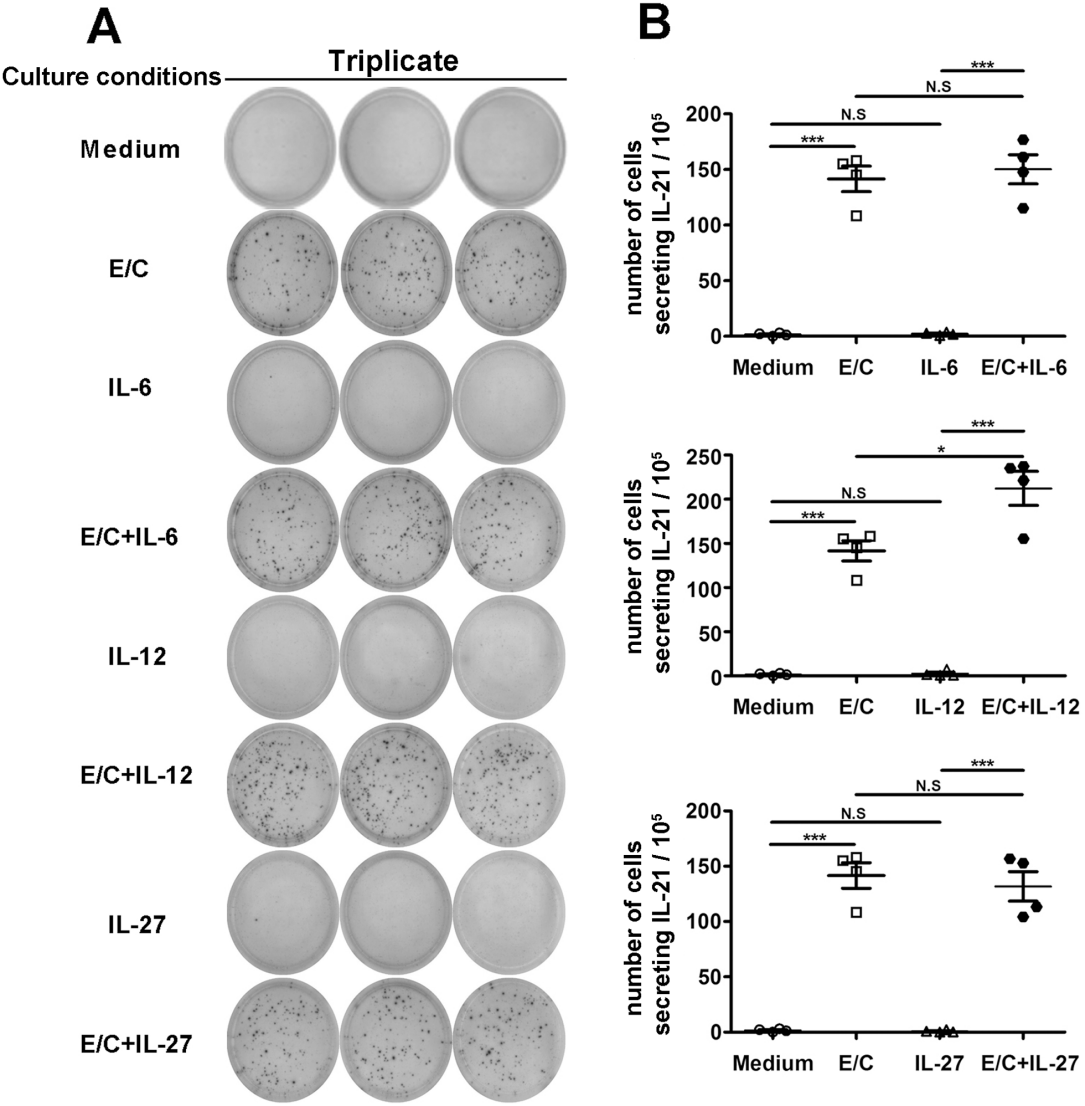
IL-12 promoted E/C peptide-induced IL-21 production. (A) PFCs were cultured in the presence of medium; with IL-6, IL-12 or IL-27 alone; with E/C peptides; or with E/C peptides plus cytokines. The number of IL-21-producing cells was determined by ELISPOT. A representative result is shown. (B) Statistical results from four independent experiments are shown. *p<0.05; *** *p*<0.001; N.S., not significant.

**Fig 8:**
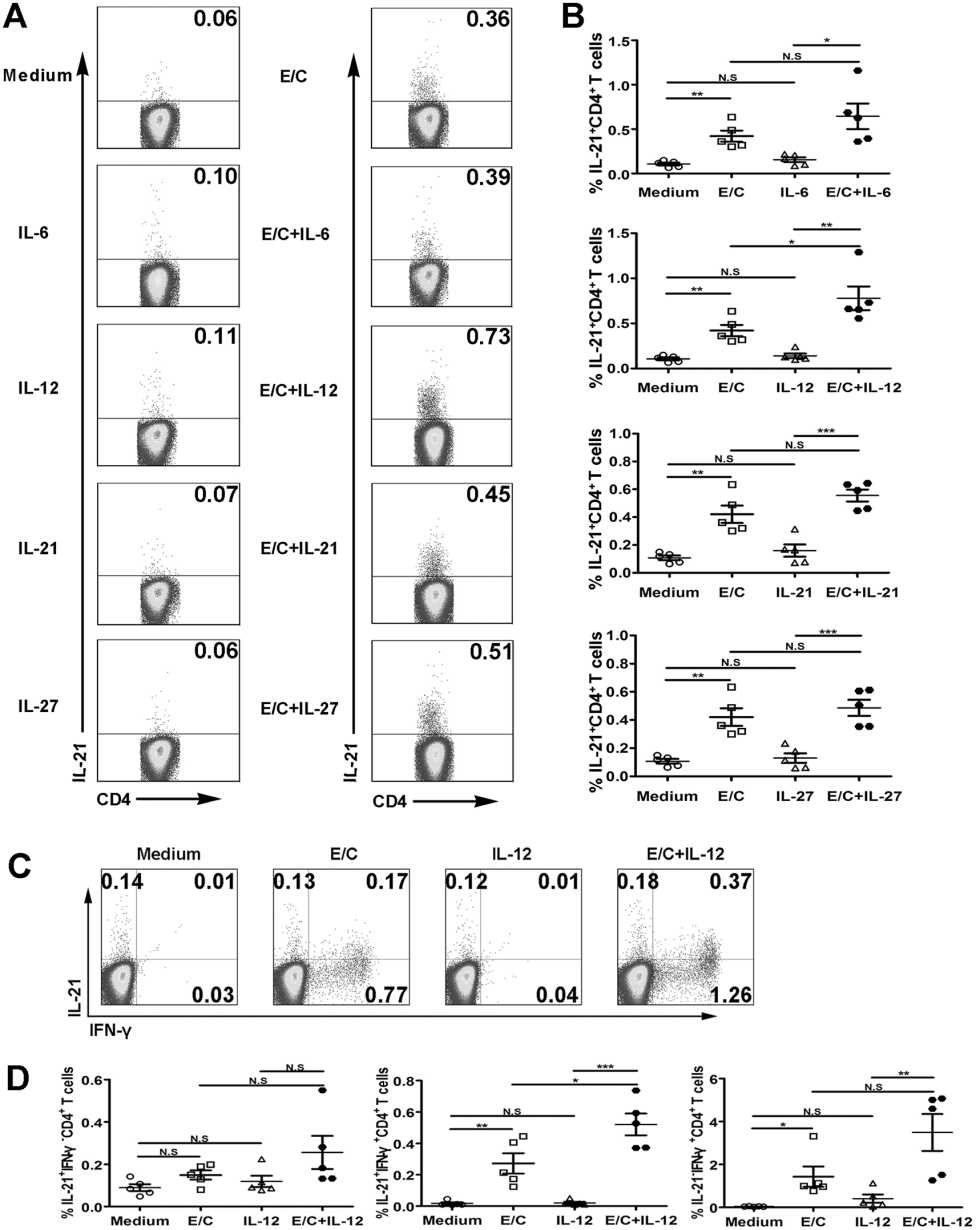
IL-12 promoted E/C peptide-induced CD4^+^IL-21^+^IFN-γ^+^ T cells. (A) PFCs were cultured in the presence of medium alone, with IL-6, IL-12, IL-21, IL-27 and E/C peptides, or with E/C peptides plus cytokines. The expression of IL-21 by CD4^+^ T cells was evaluated by FACS. Representative dot plots are shown. (B) Statistical results of IL-21 expression by CD4^+^ T cells are shown (n = 5). (C) One representative experiment showing the expression of IL-21 and IFN-γ by CD4^+^ T cells. The numbers in each quadrant represent the percentages of positive cells within CD4^+^ T cell populations. (D) Statistical results of the proportions of IL-21^+^IFN-γ^-^, IL-21^+^IFN-γ^+^ and IL-21^-^IFN-γ^+^ within CD4^+^ T cells (n = 5). *p<0.05; **p<0.01; *** *p*<0.001; N.S., not significant.

To further determine whether cytokines regulate MTB-specific IL-21 production by PFCs, an ELISPOT assay was conducted. No or few IL-21^+^ spots were formed without stimulation or in the presence of cytokine alone. However, E/C peptides or E/C peptides plus cytokine elicited strong antigen-specific IL-21 production (Fig 7A). Importantly, more IL-21^+^ spots were formed following E/C peptides plus IL-12 stimulation than stimulation with E/C peptides alone. However, IL-6 and IL-27 did not have an effect (Fig 7B). To confirm these results, we conducted FACS. Consistent with the ELISPOT results, medium and cytokine alone did not induce IL-21 production. However, both the E/C peptides and the E/C peptide plus cytokine conditions induced IL-21 production (Fig 8A). Significantly higher levels of IL-21 were induced by the combination of IL-12 and E/C peptides when compared with E/C peptides alone (Fig 8B). To specifically analyze which cell subset was regulated, the expression of IL-21 and IFN-γ was analyzed on the CD4^+^ T cell gate (Fig 8C). Analysis of cytokine-expressing CD4^+^ T cells indicated that the subset of IL-21^+^IFN-γ^+^ cells was markedly enhanced following the addition of IL-12 compared with E/C peptides alone, but no significant difference was observed with respect to the subset of IL-21^+^IFN-γ^-^ or IL-21^-^IFN-γ^+^ cells (Fig 8D). Taken together, these results indicated that IL-12, but not IL-6, IL-27, or IL-21, promotes MTB-specific IL-21 production by PFCs.

## Discussion

It is well known that MTB-specific CD4^+^ T cells are critical for protection against TB [28]. In the past few years, we published our efforts in determining the phenotype, function, and regulation of MTB-specific CD4^+^ T cells. Currently, we found that IL-21 was induced by PFCs at both the mRNA and protein level after stimulation with dominant peptides of E/C. The further studies showed that E/C peptides induced significantly higher levels of IL-21 expression in CD4^+^ T cells than in CD8^+^ T cells. In concordance with previous work [21], our study demonstrated that IL-21 was predominantly expressed by CD4^+^ T cells. There is evidence that other cell subsets can synthesize IL-21 under specific circumstances. Elevated frequencies of IL-21-compent CD8^+^ T cells have been described in HIV-1 infection and autoimmunity [29,30,31]. Our findings here were consistent with our earlier studies in nasal polyps that IL-21 could be produced by CD8^+^ T cells after PMA plus ionomycin stimulation [32]. Instead, Williams *et al*. have indicated that IL-21 production by CD8^+^ T cells was associated with high levels of activation [33]. Moreover, other studies have found that IL-21 is produced by NKT cells [22]. Recently, our studies have also demonstrated for the first time that IL-21 could be produced by NKT cells in TB infection following stimulation with MTB-specific antigens [34]. However, little data exists characterizing the production and regulation of IL-21-expressing CD4^+^ T cells during tuberculosis. These discrepancies of cell sources of IL-21 production might be due to differences in species, stimuli, or stimulation conditions.

Next, we examined the correlation of IL-21 with other cytokines, and we found that the subset of MTB-specific IL-21-expressing CD4^+^ T cells was different from the Th1, Th2, and Th17 subpopulations. A fraction of IL-21-expressing cells simultaneously produced Th1 cytokines, but not Th2 or Th17 cytokines. In support of these results, environmental factors that change the ratio of Bcl-6 to T-bet in Th1 cells may cause variability in Th1 and Tfh-like gene-expression patterns [35,36]. After stimulation with peptides, CD4^+^ T cells could simultaneously produce IFN-γ, IL-2, TNF-α and IL-21. Furthermore, IL-21^+^IFN-γ^+^CD4^+^ T cells exhibited obviously polyfunctionality than IL-21 single-expressing CD4^+^T cells.

It is well known that IL-21 is a marker for Tfh cells [24,26]. However, IL-21-producing cells cannot be classified as Tfh cells because other cell types can express substantial amounts of IL-21. In addition to the expression of IL-21, some important markers, such as ICOS, CXCR5, PD-1, as well as Bcl-6, are also critical in the identification of Tfh cells [24,26,27]. Therefore, we examined the phenotype of CD4^+^IL-21^+^ T cells. Our study showed that, in concordance with Tfh cells, MTB-specific IL-21-expressing cells expressed significantly lower levels of CD62L and CCR7, suggesting that they might migrate from secondary lymphoid organs. In addition, CD4^+^IL-21^+^ T cells expressed high levels of CD45RO, displaying the phenotype of effector vs. effector memory T cells. Although data in humans suggests that resting memory Tfh cells exist in the peripheral blood as CD45RO^+^CXCR5^+^ cells, this remains a contentious issue that requires more examination [37,38]. Recently, decreased frequencies of CD4^+^CXCR5^+^ T helper cells were reported in the blood of active pulmonary TB patients when compared with the blood of latent TB patients [39]. Moreover, Slight *et al*. have shown that CD4^+^CXCR5^+^ T cells play a protective role in the immune response against TB [40]. Nevertheless, the expression of CXCR5 is not an essential marker to define “Tfh-like” cells. For example, CD4^+^ T cells primed with IL-12 can induce B cells to produce Igs, which is dependent on IL-21 and ICOS. Thus, these cells shared fundamental characteristics with Tfh cells and can be called “IL-21-expressing Tfh-like cells”. Moreover, expression of high levels of CXCR5 leads to the migration of Tfh cells to CXCL13^+^ B-cell follicles [37,41]. Therefore, though the expression of CXCR5 was similar between IL-21^+^ and IL-21^-^CD4^+^ T cell subsets in our study, we speculate that the expression level of CXCR5 changes during cell migration to local sites. PD-1, another marker of Tfh cells, is induced by extended TCR signaling and is also high on most activated CD4^+^ and CD8^+^ T cells [24]. That might explain why PD-1 was similar between IL-21^+^ and IL-21^-^CD4^+^ T cell subsets. In addition, MTB-specific IL-21-expressing cells expressed high levels of CD40L and ICOS, suggesting that they were in an activating state, although the expression of CD25 was not that high. Taken together, our results indicated that our CD4^+^IL-21^+^ cell population corresponded to a CD45RO^+^CD62L^low^CCR7^low^CD40L^high^ ICOS^high^ cell population.

Bcl-6 is the most important transcription factor for Tfh cells [24]. Therefore, we examined the expression of Bcl-6. In concordance with the distinguishing feature of Tfh cells, Bcl-6 expression was significantly higher in CD4^+^IL-21^+^ cell population than CD4^+^IL-21^-^ T cells. Altogether, the findings indicated that MTB-specific IL-21-expressing CD4^+^ T cells having the features of Tfh cells.

Previous work has suggested that IL-21, IL-6, IL-12 and IL-27 participate in Tfh cell development and IL-21 expression [24,42,43]. Few reports have studied the regulation of antigen-specific IL-21 production by memory CD4^+^ T cells. Therefore, we examined the regulation of MTB-specific IL-21 expression. The results indicated that IL-12 up-regulated IL-21 expression induced by MTB-specific peptides. IL-21, IL-6, and IL-27 had no effect on the production of IL-21. Similarly, two independent studies have shown that IL-12 induces the generation of human Tfh-like cells from naive CD4^+^ T cells and triggers a significant increase in IL-21 expression *in vitro* [25,44]. Moreover, IL-12, which signals via STAT4, can be an early inducer of the Tfh cell phenotype, while both IL-21 and IL-6, which signal via STAT3, are insufficient to drive Tfh differentiation [45,46]. Hence, the regulation of MTB-specific IL-21-expressing cells was similar to that of Tfh cells to some extent.

IL-21, produced by Tfh cells, plays a critical role in B-cell proliferation, class switching and Ig production [24,26]. Our study showed that IL-21 up-regulated the secretion of Ig by activated PFCs (data not shown). Therefore, we speculated that CD4^+^IL-21^+^ cells shared functional properties with Tfh cells from secondary lymphoid organs. In concordance with Tfh cells, the cells required activation to provide help to B cells and induce their differentiation into Ig-producing cells.

In summary, our studies provided important data concerning the phenotype, regulation and functional capacity of MTB-specific IL-21-expressing CD4^+^ T cells in PFCs from TB pleurisy. These findings showed that MTB-specific IL-21-expressing CD4^+^ T cells displayed a CD45RO^+^CD62L^low^CCR7^low^CD40L^high^ICOS^high^ phenotype and expressed high levels of Bcl-6. All of the above characteristics are distinguishing features of Tfh cells. However, in contrast to Tfh cells, these cells did not localize within B-cell follicles [47], so it is still challenging to address whether MTB-specific IL-21-expressing cells are circulating Tfh cells or “Tfh-like” cells. Nevertheless, evaluation of MTB-specific IL-21-expressing CD4^+^ T cells could help us understand cellular and humoral immune responses to MTB.

## Materials and Methods

### Ethics statement

Informed written consent was obtained from all patients. The study protocol was approved by the Ethics Committee of the Zhongshan School of Medicine, Sun Yat-sen University (Guangzhou, China) and the Chest Hospital of Guangzhou (Guangzhou, China).

### Study participants

Thirty patients infected with *Mycobacterium tuberculosis* (12 females and 18 males, age 23 to 74 years old) were recruited into the study. All patients were newly diagnosed with TB pleurisy at the Chest Hospital of Guangzhou, China. Diagnosis of the patients was confirmed by the following criteria: positive cultures for MTB in cultures of pleural biopsy specimens or pleural fluid smear, histological evidence in biopsy specimens of pleural tissue, and positive staining for MTB. Patients who had been previously diagnosed with HIV, hepatitis B, or hepatitis C, or who had a history of autoimmune diseases, were excluded from the study. All pleurisy samples were collected before the initiation of anti-tuberculosis treatment.

### Antigens and mAbs

We selected six highly immunogenic peptides, which are largely human leukocyte antigen (HLA)-DR-restricted. Four peptides were derived from ESAT-6 and two were from the CFP-10 protein of MTB. Synthetic peptides were obtained from Shenzhen Hanyu manufacture, Shenzhen, China. The amino acid sequences of the peptides were previously described [11,12]. Lyophilized peptides were reconstituted in DMSO and stored at -80°C. Anti-CD28 (clone CD28.2) and anti-CD49d (clone 9F10) mAbs were purchased from BD Biosciences Pharmingen (San Jose, CA). The following mAbs were purchased from BD Pharmingen (San Jose, CA, USA) and were used for phenotypic, intracellular cytokine, and transcription factor analysis: CD3-PE-cy7 (SK7), CD3-PE-CF594 (UCHT1), CD4-APC-Cy7 (RPA-T4), CD4-APC (RPA-T4), CD4-FITC (RPA-T4), CD8-APC (RPA-T8), CD8-FITC (RPA-T8), IFN-γ-PE-cy7 (4S.B3), IL-21-PE (3A3-N2.1), TNF-α-PE-Cy7 (MAb11), IL-2-APC (MQ1-17H12), IL-4-PerCP-cy5.5 (8D4-8), IL-4-APC (8D4-8), IL-10-APC (JES3-19F1), CD45RO-FITC (UCHL1), CD62L-PE (Dreg56), CCR7-PE-cy7 (3D12), CD40L-PE (TRAP1), CD25-FITC (M-A251), CXCR5-Alexa Fluor647 (RF8B2), CXCR5-Alexa Fluor488 (RF8B2), PD-1-PE-cy7 (EH12.1), Bcl-6-Alexa Fluor647 (K112-91) and Bcl-6-PE-CF594 (K112-91). IL-21- Alexa Fluor647 (3A3-N2), IL-17-FITC (eBio64DEC17) and ICOS-APC (ISA-3) were purchased from eBioscience (San Diego, CA, USA). IFN-γ-FITC (45.15) was purchased from Beckman Coulter (Fullerton, CA), and IL-22-APC (142928) was purchased from R&D Systems (Minneapolis, MN, USA).

### Preparation of PFCs

Pleural fluid was obtained by thoracocentesis from tuberculosis patients. PFCs were isolated by lysing erythrocytes with an ammonium chloride solution and resuspended to a final concentration of 2×10^6^ cells/mL in complete RPMI-1640 medium (Invitrogen, Grand Island, NY, USA) supplemented with 10% heat-inactivated fetal calf serum (FCS; Sijiqing, Hangzhou, China), 100 U/mL penicillin, 100 μg/mL streptomycin, 2 mM L-glutamine, and 50 μM 2-mercaptoethanol (all from Gibco BRL).

### Cell culture conditions

PFCs were resuspended in complete RPMI-1640 medium, stimulated with 2 μg/mL peptides plus 1 μg/mL anti-CD28, 1 μg/mL anti-CD49d mAbs, or PMA (20 ng/ml; Sigma Aldrich, Saint Louis, MO, USA) plus ionomycin (1 μg/mL; Sigma-Aldrich). PFCs were cultured in the presence of medium alone, IL-6 (30 ng/mL; Peprotech), IL-12 (5 ng/ml; eBioscience, Santiago, Chile), IL-21 (50 ng/mL; Peprotech), IL-27 (40 ng/ml; eBioscience) alone, E/C peptides, or E/C peptides plus cytokines.

### PCR for IL-21

PFCs were stimulated as described above for 12 h. Total RNA was isolated using an RNeasy mini kit (Qiagen, Valencia, CA), and residual DNA was removed using RNase-free DNAse (Qiagen). Reverse transcription of total RNA to cDNA was performed at 37°C using a Reaction Ready-First Strand cDNA Synthesis kit (Promega). Amplification of cDNA was conducted in a DNA thermal cycler (Biometra, Germany) using the following conditions: 95°C for 45 s, 55°C for 45 s, and 72°C for 45 s for glyceraldehyde 3-phosphate dehydrogenase (GAPDH) and 95°C for 45 s, 51°C for 45 s, and 72°C for 45 s for IL-21. PCR was repeated for 35 cycles for both GAPDH and IL-21. Primer sequences were as follows: IL-21 sense 5’- GAG-TGG-TCA-GCT-TTT-TCC-TGT-T-3’, IL-21 anti-sense 5’-AGG-AAT-TCT-TTG-GGT-GGT-TTT-T-3’, GAPDH sense 5’-GCA-TGG-CCT-TCC-GTG-TCC-3’, and GAPDH anti-sense 5’-TGA-GTG-TGG-CAG-GGA-CTC-3’.

### Cytokine ELISPOT assay

The frequency of IL-21-producing cells was quantified by ELISPOT using a commercially available set (Mabtech). Briefly, PFCs were stimulated as described above and added to microwells in triplicate and incubated for 24 h at 37°C in a 5% CO_2_ incubator. Spot-forming cells (SFC) were enumerated using an ELISPOT image analysis system (Champspot II, Sage Creation, Beijing, China). The average of spots in triplicate wells was calculated as SFC/ 1×10^5^ PFCs.

### Flow cytometry

PFCs were stimulated with peptides as described above for 12 h in the presence of brefeldin A (BFA, 10 μg /mL; Sigma-Aldrich, St Louis, MO) for the final 8 h, and PFCs were stimulated with PMA plus ionomycin in the presence of BFA for 6 h. After stimulation, the cells were washed with PBS buffer containing 0.1% BSA and 0.05% sodium azide (FACS buffer). The cells were incubated with mAbs at 4°C for surface staining, washed twice, and fixed with 4% paraformaldehyde. The cells were then permeabilized with PBS containing 0.1% saponin and stained for intracellular cytokines. Flow cytometry was performed using BD FACSCalibur (BD Biosciences, San Jose, CA, USA) or FACSAria II (BD Biosciences, San Jose, CA, USA), and the experimental data were analyzed using FlowJo software (TreeStar, San Carlos, CA, USA).

### Statistical analysis

Data are presented as the mean ± SEM. Statistical tests were performed with GraphPad Prism software, version 5. Comparison between groups was performed by the Wilcoxon matched-pairs test (two-tailed). A value of *p*<0.05 was considered statistically significant.

### Accession numbers of genes mentioned in the manuscript

IL-21 GenBank accession no.: AF254069.
